# Study of the Warner-Bratzler Shear Force, Sensory Analysis and Sarcomere Length as Indicators of the Tenderness of Sun-Dried Beef

**DOI:** 10.3390/molecules18089432

**Published:** 2013-08-07

**Authors:** Yuri Ishihara, Ricardo Moreira, Geany de Souza, Alanne Salviano, Marta Madruga

**Affiliations:** 1 Post-Graduate Program in Food Science and Technology, Technology Centre, Federal University of Paraiba (Universidade Federal da Paraíba—UFPB), University City, João Pessoa-PB, CEP 58051-900, Brazil; E-Mails: motari@gmail.com (R.M.); alaneetamize@yahoo.com.br (A.S.); msmadruga@uol.com.br (M.M.); 2 Graduate Course in Food Engineering, Technology Centre, Federal University of Paraiba (Universidade Federal da Paraíba—UFPB), University City, João Pessoa-PB, CEP 58051-900, Brazil; E-Mail: geanytargino_@hotmail.com

**Keywords:** sun-dried beef, tenderness, sensory, shear force, sarcomere

## Abstract

Sun-dried beef is a frequently consumed and valued product in Brazil, however, there have been no scientific studies on its texture. To assess the tenderness of sun-dried beef, an instrumental analysis (Warner-Bratzler Shear Force; WBSF), a sensory analysis (Quantitative Descriptive Analysis; QDA) and the sarcomere length (SL) were used as indicators. Significant differences were observed among the sun-dried beef samples. Sample 3 (composed of sun-dried meat purchased at three fairs from Region 3 in the city of João Pessoa-PB) was considered the most tender by the assessors, with a score of 6.7, and its WBSF analysis revealed a maximum value of 2.70 kgf. Additionally, this sample exhibited the highest SL value (1.89 µm). Samples 1 and 2 (composed of sun-dried meat purchased at three fairs from Regions 1 and 2, respectively, in the city of João Pessoa) exhibited very similar tenderness values (WBSF and QDA) but differed in their SL values, which suggested that sample 2 was the least tender. In conclusion, these results demonstrate that the studied parameters are complementary and can be used as tenderness indicators for sun-dried beef. However, although the difference was beyond the detection limit of the assessors and the texturometer, the SL analysis appears to have been the most effective.

## 1. Introduction

Sun-dried beef is a characteristic product of Brazil, where refrigerated meat, typically from cattle, is lightly salted and marketed. Although it is salted, sun-dried beef has characteristics more similar to those of fresh, refrigerated beef than to those of salted products, such as charque (CH) and jerked beef (JB). Because CH and JB are considered foods with intermediate moisture content, they have water activity (Aw) values between 0.60 and 0.90 [1,2] and undergo treatments during processing, such as addition of salt and sodium nitrite, dehydration and vacuum packaging, that act as agents to provide a long shelf life (6 months) [3]. These barriers preclude the need for cold storage.

Like CH and JB, sun-dried beef undergoes salting and drying steps; however, the latter only reduces surface humidity because it is performed in the shade and for a short period of time. Thus, the shelf life of sun-dried beef under ambient conditions ranges from 2 days to 4 days [3,4], which can be somewhat extended by storing the beef under refrigeration.

The composition of charque and jerked beef is 15% to 20% salt and approximately 45% to 50% moisture. In contrast, sun-dried beef is 5% to 6% salt and 64% to 70% moisture [3]. The processing of sun-dried beef is typically artisanal. The producers, who typically produce on small scales, process the meat without any technological standardization, and these differences in processing are related to the region in which the meats are produced. Thus, there are frequent variations in the taste, color and, in particular, tenderness of commercially available products [5].

Studies on beef tenderness are common because tenderness is a determining factor in the acceptability of the product by consumers. Indicators, such as sensory evaluation and instrumental analysis by shear force, have been reported in several studies [6,7,8,9,10,11]. The sarcomere length is also frequently studied and has been shown to be related to beef tenderness [12,13,14,15,16,17].

However, no research has been undertaken to specifically assess the tenderness of sun-dried beef. Thus, this study aims to fill this information gap by using instrumental, sensory and sarcomere length evaluations as indicators of the tenderness of this meat product.

## 2. Results and Discussion

### 2.1. The Chemical Composition of Sun-Dried Beef

Differences were observed in the compositions of the studied sun-dried beef samples (Table 1). Variations in the moisture, protein, ash and sodium chloride contents were found, suggesting a lack of standardization in the processing of sun-dried beef, in agreement with previous research on the chemical composition of sun-dried beef sold in a local market [18,19]. These studies [18,19], together with the data obtained herein, suggest a sun-dried beef production in which salting and drying appear to be milder than previously observed by [1]. As a result, there are lower levels of sodium chloride, so no desalting is needed prior to consumption. There are also higher moisture content and water activity compared with other studies. This fact supports the assumption that sun-dried beef is a product without any specifications related to standards of identity and quality, thus explaining the heterogeneity of products exposed to marketing and demonstrating the need for legislation on this product.

The results displayed in Table 1 affirm that sun-dried beef, despite being a salted product, differs in terms of its composition from charque and jerked beef, which have moisture values ranging from 45.96% to 46.4% and 51.17% to 52.1%, respectively [20,21,22]. A similar observation was made when we measured higher Aw levels for sun-dried beef than those reported for charque and jerked beef, which range from 0.70 to 0.78 [3]. Additionally, the neutral pH, high water activity levels and low sodium chloride levels for sun-dried meats, compared with those of salty products, confirm a short shelf life for sun-dried meats because all of these parameters contribute to microbial growth.

### 2.2. Tenderness Study

The results from the WBSF analysis of the sun-dried beef samples, described in Table 1, demonstrate that the tenderness of samples 1 and 2 did not differ statistically. However, according to the criteria established by Bellew *et al.* [11], beef cattle carcass muscles may be classified by their shear force as: (i) very tender, SF values less than 3.2 kgf; (ii) tender, SF values between 3.2 kgf and 3.9 kgf; (iii) intermediate, SF values between 3.9 kgf and 4.6 kgf; and (iv) hard, SF values above 4.6 kgf. In this study, sun-dried beef sample 1 was classified as intermediate, sample 2 was classified as hard, and sample 3 was classified as very tender.

While developing a product similar to sun-dried beef, with the addition of 4% sodium chloride, Carvalho Jr. [5] obtained a value of 4.16 kgf for the shear force, similar to that observed for samples 1 and 2. Sousa [23] obtained higher values (6.40 kgf) when processing another product similar to sun-dried beef that included 3% sodium chloride and that was treated with sodium lactate and sodium diacetate. The added products appear to have negatively affected the tenderness of the processed beef.

While investigating the effect of maturation in the processing of sun-dried beef, intermediate values, compared with the present study (ranging from 3.08 kgf to 3.51 kgf), were obtained [18].

The sensory panel identified differences among the sun-dried beef samples, as shown in Table 1. Sample 3 exhibited a higher average score than the other samples. The following values were determined by using the hedonic scale to classify beef texture: 0 is “hard”, 4.5 is “tender”, and 9.0 is “very tender”. Samples 1 and 2 were not considered tender because they exhibited average values below 4.5. Therefore, they were characterized as hard samples. An average value of 3.5 [18] was obtained when using a QDA analysis with a panel of nine assessors and the same hedonic scale as in the present study. This value is similar to the averages obtained in the present study for sun-dried beef samples 1 and 2, suggesting that the origin of the beef may have been the same.

A significant correlation (−0.69; *p* < 0.01) was observed between the WBSF and the QDA. A strong correlation (−0.80; *p* < 0.05) between the SF and the tenderness values, which were assigned by a trained panel, was observed for the samples that were removed in a direction parallel to the *longissimus* muscle fiber axis [24]. While assessing consumer ability to discern different levels of beef tenderness using the WBSF analysis [6], a strong correlation (−0.72; *p* < 0.01) between the shear force and the consumer response was observed. Other studies have also linked the sensory attributes reported by a trained panel of assessors with the Warner-Bratzler shear force [25,26].

The most desirable sarcomere shortening is a percentage decrease on the order of 0% to 40% relative to the length under resting conditions [27]. Based on an average length of 1.82 µm for topside beef muscles (*semimembranosus* and *adductor femoris*) [14], values below 1.09 µm would indicate hardness. Thus, all the sarcomere lengths of the analyzed sun-dried beef samples indicated good tenderness levels. Nevertheless, emphasis is given to sample 3, which appeared to be the most tender because it had the highest sarcomere length.

These results are consistent with the results for the sarcomere length of the *semimembranosus* post-rigor, in which an average value of 1.82 µm was observed for 18 sarcomeres [14]. These results are also in agreement with a study that investigated the relation between the sarcomere length and the tenderness of the bovine *longissimus* muscle, in which an average value of 1.79 µm was observed for 15 analyzed sarcomeres [12]. In another study, an average value of 1.80 µm was observed for the sarcomere length of the *longissimus* and *semimembranosus* muscles, and 1.90 µm was observed for the *adductor* muscle [17].

**Figure 1:**
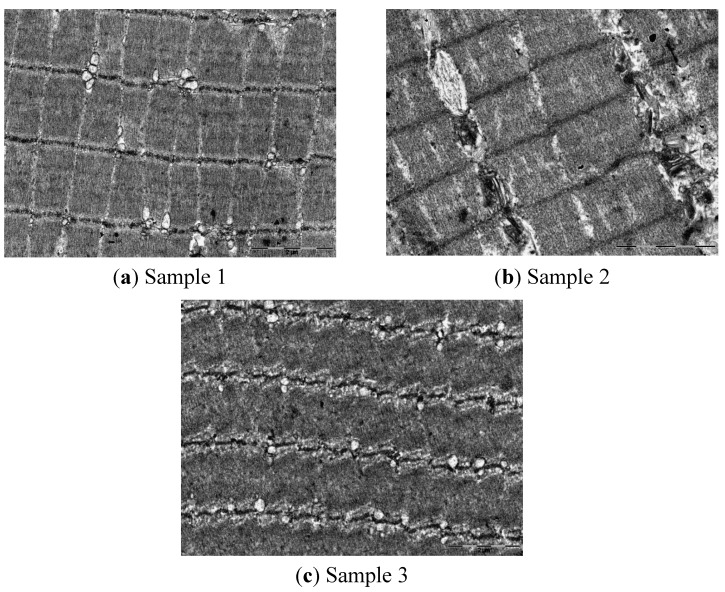
Sun-dried beef micrographs. Longitudinal sections of myofibrils. Magnification: (**a**) Sample 1 **(**11,000×); (**b**) Sample 2 (14,000×); (**c**) Sample 3 (8,900×).

In the micrographs shown in Figure 1, a pattern of bands that is characteristic of muscle fibers formed by sarcomeres (the structural unit between the Z-discs) can be observed. In the three samples, the muscle fibers showed shrinkage, which was likely due to the use of cold temperatures in meat preservation. Clear (I) or dark (A) bands are clearly visible, however, the M lines are not clear between the A and I bands. The Z lines appear to be fragmented in samples 1 and 3. In sample 3 (Figure 1c), the Z-discs appear to be more degraded than in samples 1 and 2, suggesting differences in the tenderness among the samples.

Tenderness in meat products is the result of multiple factors, including muscle contraction, protease activities and both fat and moisture content [22]; however, the results obtained in this study have shown that some analytical tools can help to identify differences between the tenderness of samples under study. Sensory analysis, shear force and sarcomere length efficiently indicated that one of the samples (sample 3) was the softest. However, the sarcomere length analysis proved to be the most accurate because it could detect differences between the other samples. It is noteworthy that instrumental and sensory analyses should be performed jointly to provide personal and instrumental responses that contribute to the decision-making process when performing investigative work.

**Table 1:** Physicochemical qualities and tenderness of the sun-dried beef.

Parameters ^1^	Sample 1	Sample 2	Sample 3
Moisture (g/100 g)	74.28 ± 0.21 ^a^	71.14 ± 0.13 ^b^	70.52 ± 0.04 ^c^
Protein (g/100 g)	21.86 ± 0.09 ^c^	23.73 ± 0.16 ^a^	22.64 ± 0.20 ^b^
Lipids (g/100 g)	0.53 ± 0.02 ^a^	0.35 ± 0.12 ^a^	0.52 ± 0.10 ^a^
Ash (g/100 g)	4.40 ± 0.09 ^c^	5.09 ± 0.13 ^a^	4.63 ± 0.05 ^b^
Sodium chloride (g/100 g)	3.24 ± 0.12 ^b^	4.92 ± 0.12 ^a^	2.90 ± 0.06 ^c^
Aw (26.4 °C)	0.97 ± 0.06 ^a^	0.94 ± 0.06 ^b^	0.96 ± 0.06 ^a, b^
pH	6.01 ± 0.06 ^a^	5.92 ± 0.01 ^a^	5.87 ± 0.04 ^a^
Sarcomere length (µm)	1.78 ± 0.10 ^b^	1.40 ± 0.10 ^c^	1.89 ± 0.17 ^a^
WBSF (Kgf) ^2^	4.48 ± 0.89 ^a^	4.79 ± 0.84 ^a^	2.70 ± 0.79 ^b^
QDA (average score) ^2^	3.8 ^b^	2.8 ^b^	6.7 ^a^

^1^^.^ Means with the same superscript letters in the same row indicate no significant difference (*p* > 0.05); ^2^^.^ These analyses were performed with cooked samples.

## 3. Experimental

### 3.1. Sun-Dried Beef Sampling

A quantitative survey was conducted on the marketing of sun-dried beef in all fairs in the city of João Pessoa, which are traditional places that sell this product. The city was divided into three regions (R1, R2 and R3) in which three fairs with the highest sale volumes of sun-dried beef were surveyed. Samples 1, 2 and 3 were from sun-dried beef purchased at three fairs from Regions 1, 2 and 3, respectively.

All sun-dried beef samples were processed from *semimembranosus* and *adductor femoris*, which, in Brazil, are popularly known as *coxão mole*, from zebu breeds (4 to 5 years old) and weighed approximately 6.0 kg each. The meat was sliced (approximately 5 cm thick), salted (10% w/w using refined salt for 4 h), washed under running water, shade dried for 30 min and cooled. This sun-dried meat process was reported by traders that, in addition to marketing, also produced sun-dried beef.

### 3.2. Physicochemical Analyses of the Sun-Dried Beef

The moisture, protein, ash and sodium chloride contents of the beef were determined according to the Association of Official Analytical Chemists (AOAC) methods [28]. Total lipids were determined according to the methodology of Folch *et al.* [29]. Water activity was measured using direct readings on a Decagon AquaLab CX-2 meter, and pH readings were made with a Quimis digital pH meter, model Q-400 AS.

### 3.3. Tenderness Study

The instrumental texture of the sun-dried beef was evaluated by the shear force, which was measured using a TAXT-2i texturometer coupled with a Warner-Bratzler blade (Warner-Bratzler Shear Force; WBSF). The sun-dried beef samples were cooked according to the recommendations of the American Beef Science Association (AMSA) [30]. According to the processing conditions, there was no need to desalt the sun-dried beef due to the low salting level used. Steaks 2.5 cm thick were used. The steaks were grilled in an electric grill (Mondial, model Super g-01) with heating on the bottom and top plates. To monitor the temperature of the geometric center of each steak during cooking, a portable digital thermometer was used (Hanna model HI 145-20). The steaks were grilled and, when the temperature reached 40 °C, were turned until a final temperature of 71 °C was reached. Once cooked and cooled to room temperature, the samples were packed in sealed plastic bags and cooled at 4 ± 2 °C for 24 h. Six cylinders (diameters of 1.27 cm) were manually removed from each steak longitudinal to the muscle fibers using a punch tool adapted to an electric drill. The beef cylinders were sheared in a texturometer (was used to test speed of 5 mm/s) and the average of six readings was used as the final value (expressed in kgf).

Quantitative descriptive analysis (QDA) was used for sensory evaluation of the sun-dried beef samples. The samples were prepared according to the methodology used in the instrumental determination of the texture. The sun-dried beef samples were served to a sensory panel composed of six judges who had been previously selected and trained in three sessions.

The sensory panel initially consisted of 17 volunteers (students, researchers and staff from the Technology Centre of UFPB). The pre-selection of the candidates was performed based on their interest, availability, discriminative ability related to beef, familiarity with the product and ability to express proportionality using scales. The candidates then underwent basic taste and odor recognition tests as well as triangular tests to evaluate their discriminative ability [31].

The selection of assessors was based on their discriminative ability (sample PF < 0.50, reproducibility repetition PF > 0.05) and consensus with the panel in more than 80% of the descriptors, in accordance with the methodology proposed in [32]. Thus, a quantitative descriptive analysis was performed by the final panel of judges, which was composed of six individuals (21 to 50 years of age). Each assessor expressed their opinion of the tenderness of the beef using a structured nine-centimeter linear scale anchored at the extreme left, which indicated little tenderness (hard), with the center and extreme right positions indicating tender and very tender, respectively.

The candidates signed a consent form, as required by Resolution No. 196 of 10/10/1996, from the National Health Council [32]. This study was approved by the Ethics Committee for Human Research of UFPB under protocol No. 154/2011.

To visualize the sarcomere length, 0.5 cm^3^ cubes were removed and immersed in Karnovsky’s fixative solution, which consisted of 2.5% glutaraldehyde, 4% paraformaldehyde and 0.1 M sodium cacodylate. Each cube was cut to approximately 2 × 2 × 2 mm^3^, washed successively with cacodylate buffer to remove the fixative and post-fixed with 1% osmium tetroxide, 10 mM calcium chloride, 1.6% potassium ferrocyanide and 0.2 M sodium cacodylate buffer. The samples were then washed with 5% uranyl acetate, dehydrated with increasing concentrations of acetone and embedded in resin. Semi-thin cuts were made with an ultramicrotome (LEICA EM UC6 MZ6) to determine the direction of the muscle fibers. The blocks from each sample were then cut into ultrathin sections. The sections were treated with 5% uranyl acetate and lead citrate. For sarcomere identification, myofibril images were viewed with an FEI Morgagni 286D microscope operating at 80 kV. To determine the sarcomere length, the ITEM software installed in the FEI Morgagni 286D microscope was used. For each sample, 25 randomly chosen myofibril fragments were evaluated, and the lengths of 6 sarcomere units of each fragment were measured [33,34].

### 3.4. Statistical Analyses

Statistical analyses were performed using an analysis of variance (ANOVA), and a comparison of means was performed using Tukey’s test at 5% significance. The statistical analyses were performed with the SAS software [35]. A correlation coefficient was generated to describe the relation between the WBSF and the sensory analysis using the procedure PROC CORR [35]. 

## 4. Conclusions

Based on our results, it can be concluded that there were differences in the processing of the sun-dried meat, which is evidenced by variations in the composition of the data, indicating the need for specific legislation to standardize the processing of this product and parameters to identity the product and its quality. 

Differences in the tenderness of the samples were also observed. Although fat influences meat tenderness, tenderness did not vary with fat content in this study, likely due to the low fat content of the samples. The shear force showed variations from hard (sample 2) to very soft (sample 3). All softness indicators demonstrated that sample 3 was the softest. Samples 1 and 2 differed only in their sarcomere lengths, which indicated that sample 2 was the softest, suggesting an improved accuracy with this analysis because the length displayed a higher sensitivity than the sensory analysis and shear force. In this context, it can be inferred that the softness indicators used in this study were satisfactory and complementary when applied to sun-dried meat.
